# Two dimensions of social anxiety disorder: a pilot study of the Questionnaire for Social Anxiety and Social Competence Deficits for Adolescents

**DOI:** 10.1186/s13034-015-0079-y

**Published:** 2015-10-08

**Authors:** Carolin Fernandez Castelao, Katharina Naber, Stefanie Altstädt, Birgit Kröner-Herwig, Uwe Ruhl

**Affiliations:** Department of Clinical Psychology and Psychotherapy, Georg-August-University of Göttingen, Gosslerstr. 14, 37073 Göttingen, Germany

**Keywords:** Social anxiety disorder, Social anxiety, Social competence deficits, Adolescents, Clinical diagnostics, Questionnaire

## Abstract

**Background:**

The Questionnaire for Social Anxiety and Social Competence Deficits for Adolescents (SASKO-J) was developed as an instrument for clinical diagnostics of social anxiety disorder in youths by measuring social anxiety and social deficits in two separate dimensions. The study provides an initial assessment of the scale’s psychometric properties in a clinical sample.

**Method:**

The reliability and validity of the SASKO-J were assessed in a mixed clinical sample of 12- to 19-year-old German adolescents *(N* = 85; mean age 15.71 years; *SD* = 1.92; 62.4 % girls). In a second step, the diagnostic validity was evaluated in a clinical sample of 31 adolescent patients with social anxiety disorder (mean age 16.10 years; *SD* = 1.54; 74.2 % girls) and a sample of 115 German high school students (mean age 15.84 years; *SD* = 1.65; 60.9 % girls) via Receiver Operating Characteristic (ROC) analysis.

**Results:**

The internal consistencies of the total scale and the subscales were good to excellent (0.80 ≤ α ≤ 0.96), and the results indicated a good convergent and divergent validity. The ROC analysis revealed a satisfying area under curve (AUC = 0.866), and a cutoff of 41.5 for the SASKO-J total score represented the best balance of sensitivity (0.806) and specificity (0.826).

**Conclusions:**

The results of this pilot study provide initial support for the clinical use of the SASKO-J in the diagnostic process. Future research should address the question of psychometric properties in a social anxiety disorder sample as well as the questionnaire’s sensitivity for detecting change in symptoms during therapy.

## Background

Social anxiety disorder is one of the most challenging disorders in adolescence [1–3]. During this age, the incidence of social anxiety increases notably [4–6]. Adolescence is an important developmental stage with regard to emotional, cognitive, biological, and social changes [7, 8] where youths are confronted with many psychologically relevant challenges. For example, they have to deal with questions of identity and self-perception as well as with increasing autonomy and responsibility. At the same time, relationships with peers and romantic partners get more important and influence the development of self-esteem and social competencies [9, 10]. In addition, the significance and frequency of achievement at school and during leisure time also increases [11]. Since cognitive abilities increase in adolescence, reflections and self-evaluations become more detailed and often more critical [12]. As a consequence, the time of adolescence is characterized by high self-awareness and self-criticism and thus, can result in high vulnerability especially with regard to social anxiety and social problems [7, 13].

Symptoms of social anxiety disorder are often stable through adolescence [14, 15] and can persist into adulthood [16]. Besides the risk of chronicity, there is a large amount of accompanying psychosocial risk that can hinder the psychological, emotional, and social development of adolescents [16–19]. Youths who suffer from social anxiety disorder often have problems at school or work and difficulties that are related to interactions with peers and intimate partners [20–25]. Moreover, comorbid disorders as depression, other anxiety disorders, and/or alcohol abuse often develop [16, 26–28]. As a consequence, socially anxious youths have a lower educational level, are more often unemployed, are less socially integrated, and are less often in partnerships compared to their healthy peers [16, 25, 29]. Because of these risks, an early and adequate identification as well as an appropriate intervention for social anxiety are desirable.

Beyond the characteristic symptoms of social anxiety disorder like intensive fear and avoidance of social situations on the basis of evaluation anxiety or anxiety of being in the focus of attention [30, 31], some patients also suffer from *s*ocial competence deficits [32–35]. Social competence deficits can be unobservable, e.g., deficits in social cognition, regulation of attention, decoding and interpretation of information, empathy, and regulation of behavior, but can also be observable in motor and verbal behavior, e.g., frequency and duration of eye contact, gestures, and initialization of a conversation [36]. Adolescents with such deficits often worry about not meeting social expectations [21, 37]. Since peer relations are very important in adolescence [9], deficits in social competence might endanger important developmental progress by leading to difficulties in establishing and maintaining adequate social contacts.

Several studies have shown that youths with social anxiety disorder have less social competencies than healthy controls. These youths evaluated their own behavior worse than their peers and were rated more incompetent by independent observers. For example, Spence et al. [38] documented those children aged 7–14 years with social anxiety disorder seldom initialized social interactions, interacted less with others, and gave short answers. Inderbitzen-Nolan et al. [39] identified poorer evaluations of social anxious adolescents (12–16 years) than of their healthy partners during role play in different categories, e.g., self-confidence, social competence, and assertiveness. These results were replicated in the studies of Alfano et al. [40], Beidel et al. [41], and Miers et al. [35]. Difficulties in empathy and interpretation of facial expressions also were found in this group [40]. However, not all youths with social anxiety disorder showed social deficits [42–45]. Hence, differences in the frequency and occurrence of social competence deficits in youths with social anxiety disorder can be expected.

In clinical settings, the co-occurrence of social anxiety and social competence deficits is often observed; however, the presence of deficits is not an integral part of the diagnostic criteria for social anxiety disorder according to the ICD-10 or DSM IV [30, 31]. However, social competence deficits can maintain or exacerbate symptoms of this disorder; therefore, they should not be disregarded [36]. Since a significant relationship between the constellation of symptoms and the severity of the disorder must be assumed, an appropriate clarification about individually relevant symptoms prior to therapy is required [46]. Thus, at the beginning of therapy, it should be assessed if—and if yes, what type of—social deficits exist beyond symptoms of social anxiety. Only on this basis can an adequate and individual therapy with regard to duration, focus, and intensity of therapy be developed [47, 48], which in turn should lead to a better outcome.

Nowadays, social competence training is often provided as an optional part in manuals for the therapy of social anxiety disorder and as a consequence, is often integrated into therapy [47–49]. However, some studies have shown that such trainings are not always effective [50]. One reason for this finding can be seen in the general behavioral focus of social competence trainings [51], although different types of social problems in patients with social anxiety disorder (e.g., cognitive deficits, communication deficits, performance deficits) occur [36]. Moreover, the diagnostic basis of the decision to include or not to include a module with focus on social competence is difficult since an adequate measure of social deficits has not been available [52]. There are some well-accepted questionnaires for social anxiety disorder in youth [53, 54], for example the Social Anxiety Scale for Adolescents (SAS-A) [55], the Social Anxiety Scale for Children-Revised (SASC-R; La Greca) [55], the Social Phobia and Anxiety Inventory for Children (SPAI-C) [56], and the Social Anxiety and Avoidance Scale for Adolescents (SAASA) [57]. All of these mainly measure symptoms of anxiety, avoidant behavior, and dysfunctional cognitions; however, items regarding deficits in social competence are neglected. Thus, a questionnaire that explicitly measures such deficits and separates them from social anxiety has not yet been developed. Such an instrument would be essential for clinicians to be able to improve their decisions on whether competence training is warranted and if yes, what competencies should be emphasized. Hence, this type of instrument could improve current practices regarding therapeutic decisions.

A few years ago, Kolbeck and Maß [36] published the Questionnaire for Social Anxiety and Social Competence Deficits (SASKO) for adults as the component of deficits had also been lost [37]. The key feature of the SASKO is the separate measurement of social anxiety and social competence deficits as two distinct dimensions. The authors argued that social anxiety and social deficits interact with each other and thus cannot be regarded isolated. Rather, they should be considered as different components of social anxiety disorder [36]. This assumption aligns with the model of Wlazlo (1989; cited in Kolbeck and Maß [36]) who described social anxiety and social deficits as central components of the disorder. In addition, through the differentiation of behavioral and cognitive competencies within the deficit dimension, the SASKO allows a deeper insight into possible deficits [36].

As the SASKO has proved consistently good psychometric properties [36], it was adapted for use with adolescents (SASKO-J) [58]. The conceptual separation and the underlying five-factor structure of the questionnaire for adults (i.e., two anxiety scales, two deficit scales, and one additional scale that measures loneliness) has been confirmed for the SASKO-J [58]. The results of an unselected sample of 228 German students showed satisfactory to good consistencies (0.77 ≤ α ≤ 0.94) and retest-reliabilities (0.56 ≤ *r*_*tt*_ ≤ 0.87) for the subscales and the total scale [58]. Additionally, in a sample of 115 German students, good convergent (0.39 ≤ *r* ≤ 0.80) and divergent (0.19 ≤ *r* ≤ 0.31) validity of the SASKO-J was documented for the total scale and the majority of subscales [58]. Thus, there is strong evidence that the questionnaire can be used with adolescent samples. However, because the SASKO-J was predominantly developed for application in patients, evaluation of its feasibility and diagnostic quality in clinical samples is still lacking.

In the first step of the present pilot study, the reliability and validity of the SASKO-J was tested in a mixed clinical sample^1^ of adolescents aged 12–19 years. Since the SASKO-J is supposed to improve the diagnosing of social anxiety disorder, it is important to examine its accuracy in differentiating individuals with or without social anxiety disorder. Thus, in the second step, we tested the sensitivity and the specificity of the SASKO-J. For this purpose, a specific clinical sample was recruited consisting only of adolescent patients who suffered from social anxiety disorder. Furthermore, a sample of non-selected high school students was assessed that provided the comparison sample. On this basis, a cutoff was computed to determine the critical value that allows an accurate classification and differentiation of adolescents with and without a possible social anxiety disorder diagnosis.

With regard to the first aim and based on the results from previous studies on the SASKO-J [58], we expected good reliability (internal consistency) of the SASKO-J in the mixed clinical sample. Furthermore, we assumed good convergent and divergent validity of the SASKO-J in this sample. We expected that the anxiety scales would be more strongly associated with the convergent measurement of social anxiety disorder than the deficit scales due to their conceptual similarity. With regard to the second aim, when comparing high school students (non-clinical sample) with adolescent patients (clinical social anxiety disorder sample), we assumed that the patients would have significantly higher scores on all scales of the SASKO-J than the students. Concerning the accuracy, we expected that the questionnaire would adequately discriminate between these two groups and present high sensitivity and specificity.

## Methods

### Participants and procedure

#### Clinical samples

The recruitment of adolescent patients was conducted from spring to autumn in 2013 via contacts with several psychotherapeutic/psychiatric clinics, outpatient services, and practices in different cities in the northern part of Germany. These institutions were considered to be common clinical settings for the treatment of adolescents with psychiatric disorders (e.g., social anxiety disorder). An information letter explaining the aim of the study was sent to all institutions. Moreover, further information was offered via a personal meeting. From the 100 institutions originally contacted, only 36 answered. From these, 19 institutions agreed to participate in the study. Seventeen institutions refused participation, e.g., for reasons of expected high work load. The particular assessment procedure varied slightly over the different institutions regarding the distribution and collection of questionnaires but the basics of the procedures were equal.

The questionnaires were taken to the institutions by the research assistant or were sent via post. The therapists in the institutions handed out the questionnaire package (four questionnaires) to their adolescent patients and then collected them. The patients completed the questionnaires during their therapeutic session. In the clinical settings, group tests were administered by the research assistant. The study was accepted by the Ethic Commission of the Psychological Institute of the University of Göttingen. Every full-age adolescent received an information letter and the informed consent, which they signed after agreeing to participate in the study. Parents of minors also received an information letter and the consent form. We guaranteed the data would be anonymous and that participants could resign in any phase of the study. Each therapist was asked to complete a short data entry form for each of his or her patients (information about diagnosis, process of diagnosis, type(s) of medication, length of psychotherapy, age, language, and IQ). Adolescents were included in the study if they were between 12 and 19 years old, had a psychiatric diagnosis, sufficient knowledge of the German language, and an IQ ≥85. In addition, patients diagnosed with social anxiety disorder were excluded if they took anxiolytic drugs.

In the first step of the study, we recruited a mixed clinical sample of adolescents. The final sample consisted of 85 adolescents (*mixed clinical sample*; mean age 15.71 years, *SD* = 1.92, range 12–19 years, 62.4 % girls). Almost half of the patients were in inpatient treatment (45.9 %), about one-third (37.6 %) in outpatient treatment, and 16.5 % in day care treatment. The diagnosis was based on the ICD-10 [31]. The majority of youths had an anxiety disorder (41.2 %, thereof, 17.6 % had a social anxiety disorder) as their main diagnosis; the second largest group showed an affective disorder (25.9 %). Another large group of adolescents showed a behavioral or emotional disorder with onset in childhood and adolescence (21.2 %; e.g., ADHD, conduct disorder, separation anxiety disorder). A disorder of the schizophrenic spectrum was presented by 8.2 %, and respectively 1 % of the sample showed anorexia nervosa, substance abuse, or dysfunctional impulse control as their main diagnosis. More than half of the patients presented a comorbid disorder (55.3 %). The diagnostic procedures varied in the different institutions, but all adolescents were diagnosed on the basis of an expert opinion. Half of them were additionally diagnosed through a diagnostic interview (e.g., K-DIPS, CIDI). Almost one-third of the patients (*n* = 28) were in pharmacological treatment at the time of assessment [16 antidepressants, 10 neuroleptics, and 1 anxiolytics (this patient did not suffer from social anxiety disorder); data were available only for *n* = 27]. Almost all adolescents (*n* = 84) attended psychotherapy; however, no statement about mean duration of therapy can be given as data was only available from half of the sample (50 %). Inpatients, outpatients, and patients in day care did not differ significantly with regard to age (0.39 < *p* < 0.55), sex (0.10 < *p* < 0.68), main diagnostic categories (anxiety disorders, affective disorders, disorders with onset in childhood; 0.06 < *p* < 0.75) or with regard to comorbidity (0.14 < *p* < 0.52). More inpatients than outpatients were in pharmacological treatment (*p* < 0.001), but the other groups did not differ significantly in this regard (*p*s ≥ 0.11).

Because only 15 of the 85 patients were diagnosed with social anxiety disorder in the mixed clinical sample, we restarted the recruitment to enlarge this subsample for our second aim. The procedure was comparable to that of the previous one. Finally, a total sample of 31 youths with social anxiety disorder was available (*social anxiety disorder sample*; mean age 16.1 years, *SD* = 1.54, range 12–19 years, 74.2 % girls). The majority (74 %) had social anxiety disorder (F40.1) as their main diagnosis and 10 % as a secondary. Five additional adolescents (16 %) had the diagnosis of social anxiety in childhood (F93.2). These youths are also referred to as socially anxious patients. Most of these adolescents (65 %) had a comorbid disorder. All therapists based the diagnosis on their expertise. In addition, more than half of the therapists (58.11 %) based their diagnoses on diagnostic interviews (e.g., K-DIPS, CIDI). Half of the sample was inpatients (51.6 %), more than one-third (38.7 %) were outpatients, and 9.7 % were in day care. The majority of adolescents (*n* = 29) were treated by psychotherapy with a mean duration of 29.62 weeks (*SD* = 36.09). Nine patients also underwent pharmacological treatment (7 antidepressants, 1 neuroleptic, and 1 psychostimulant).

#### Student sample

The procedure for the sample of students was similar to that of the clinical sample; however, we adapted the information letters and letters of agreement for this sample. This part of the study was also accepted by the Ethic Commission of the Psychological Department of the University of Göttingen. The nonclinical sample was recruited from two schools in northern Germany. Teachers and students in grades 7th to 11th were informed about the research project by the research assistant and were asked for cooperation. Students and their parents received an informed consent form. All students (*n* = 118) returned the signed consent form to their teachers. The package of four questionnaires was given to them one week later. After completing the questionnaires at home, they returned them to their teachers. Three students had to be excluded: one was out of the age range and two questionnaires were rated as invalid. The final sample consisted of 115 students (mean age 15.84, *SD* = 1.65, range 12–19 years, 61 % girls). The majority of students attended a grammar school^2^ (46 %) or a comprehensive school (44.3 %), whereas about 10 % visited a secondary general school or secondary modern school.

### Measures

#### SASKO-J

The Questionnaire for Social Anxiety and Social Competence Deficits for Adolescents (SASKO-J) [58] is a German-language self-report measure for adolescents aged 12–19 years. It differentially measures social anxiety and social deficits. It consists of 44 items (4-point scale; 0 = never to 3 = always) that represent five factors. The two anxiety scales focus on *fear of talking and fear of being in the focus of attention* (TALK; 12 items: e.g., “I get nervous when I am the focus of attention”) and *fear of rejection* (REJECT; 10 items: e.g., “For me it is hard to make a fool of myself”). Two deficit scales include *interaction deficits* (INTERAC; 10 items: e.g., “For me it is difficult to have a casual conversation with others”) and *information-processing deficits* (INFORMAT; 8 items: e.g., “I don’t know how others see my behavior”). One additional scale measures *loneliness* (LONELY; 4 items: e.g., “I suffer from having little contact with others”). The LONELY scale is excluded from the total scale (40 items). The psychometric properties in a sample of German students were satisfying [58].

#### Validity measures

For the evaluation of the convergent validity, the German version of the Social Phobia and Anxiety Inventory for Children (SPAIK) [59] was used. It is an established self-report measurement consisting of 26 items that measure cognitive, behavioral, and somatic symptoms in the context of social anxiety (3-point scale; 0 = never to 2 = always). The items mainly focus on interaction and achievement situations. The SPAIK shows a good internal consistency and retest reliability as well as a good convergent and factorial validity [59, 60]. In the current study, the internal consistency of the total scale was very high in the mixed clinical sample (α = 0.96).

For the examination of the divergent validity, the German version of the Youth Self Report (YSR) [61] was used. It is a well-established self-report questionnaire for behavioral, emotional, and somatic symptoms in youths. The original version includes 112 items (3-point scale; 0 = not true to 2 = usually true), which can be divided into eight syndrome scales. Due to economic reasons, a shortened version of the YSR (YSR-K) was used in the present study. The YSR-K consists of 32 items and only a total score was calculated. Its psychometric properties had not been evaluated previously. However, the YSR mainly matches with the Child Behavior Checklist (CBCL) [62] as the corresponding parental questionnaire. The short form of the CBCL has been consistent in its applicability. Lemanek et al. [63] found good internal consistency for the internal and external scale. In addition, a satisfying relationship between the original and the shortened form was demonstrated [63]. In the present study, the internal consistency of the total score of the YSR-K in the mixed clinical sample was high (α = 0.88).

The Depression Inventory for Children and Adolescents (DIKJ) [64] served as an additional instrument for the evaluation of divergent validity. The DIKJ is a self-report questionnaire for the measurement of depressive symptoms in youths. It consists of 26 items and measures the occurrence of different symptoms on a 3-point scale (0 = symptom is non-existent to 2 = symptom is highly pronounced). The questionnaire has good psychometric properties [64]. In the present study, the internal consistency in the mixed clinical sample was very high (α = 0.91).

### Statistical analyses

The statistical analyses were conducted using SPSS 20 for Windows. In all analyses, missing values were replaced by means. This procedure is acceptable as the frequencies of missing values in all samples were below 5 %. With regard to the validity measures, the mean scores of the total scales were used. The additional scale, LONELY, of the SASKO-J was considered in all analyses; however, in accordance to the procedure of Kolbeck [46] and Fernandez Castelao [58], it was not included in the computation of the total scale.

## Results^3^

### SASKO-J^4^: Descriptive data and reliability

The means, standard deviations, mean item difficulties, indices of selectivity, and internal consistencies of the subscales of the SASKO-J are presented in Table 1. The internal consistency of the total scale was very high (α = 0.96) and the subscales showed good to very good reliabilities (0.80 ≤ α ≤ 0.91). The relationships among the subscales showed moderate relationships (between *r* = 0.42 and *r* = 0.65); their correlations with the total scale were higher (0.50 ≤ *r* ≤ 0.81).

**Table 1:** Characteristics and reliabilities of the subscales and the total scale of the SASKO-J

Scale	*M* (range)	*SD*	*P*	*r*_*it (i)*_	α
TALK	13.80 (0–31)	7.65	38.25	0.83	0.91
REJECT	12.10 (0–28)	6.95	40.20	0.78	0.90
INTERAC	8.87 (0–24)	5.74	29.50	0.82	0.85
INFORMAT	7.76 (0–17)	3.98	32.13	0.77	0.80
LONELY	3.19 (0–10)	3.01	26.57	0.66	0.83
TOTAL	42.52 (0–91)	21.82	35.02	0.80	0.96

Results are based on the mixed clinical sample (*N* = 85)

*M* mean, *SD* standard deviation, *P* mean item difficulty, *r*_*it(i)*_ mean selectivity (part-whole corrected), *α* Cronbach’s alpha, *TALK* scale “fear of talking and fear of being in the focus of attention”, *REJECT* scale “fear of rejection”, *INTERAC* scale “interaction deficits”, *INFORMAT* scale “information-processing deficits”, *LONELY* scale “loneliness”, *TOTAL* total scale

### Validity of the SASKO-J

The total scale and all subscales of the SASKO-J were significantly associated with the SPAIK total score (all *p* ≤ 0.01, see Table 2). The two anxiety scales TALK and REJECT showed very good (*r* = 0.80 and 0.90) convergent correlations. The relationships with the two deficit scales INTERAC and INFORMAT were lower (*r* = 0.76 and *r* = 0.68) but they still exceeded the critical value of *r* = 0.60 that characterizes a coefficient as satisfactory for convergent correlations [65]. The significant correlation with the additional scale, LONELY, was below the critical value (*r* = 0.54). To examine if the correlation coefficients of the anxiety and deficit scales differed significantly from each other, we calculated Fisher-Z transformations. All comparisons differed significantly with higher correlations for the anxiety scales (REJECT-SPAIK vs. INFORMAT-SPAIK: *z* = 1.887, *p* = 0.049; TALK-SPAIK vs. INTERAC-SPAIK: *z* = 3.560, *p* < 0.001; TALK-SPAIK vs. INFORMAT-SPAIK: *z* = 4.650, *p* < 0.001) except for the comparison REJECT-SPAIK vs. INTERAC-SPAIK (*z* = 0.618, *p* = 0.536).

**Table 2:** Correlations between the SASKO-J subscales and the validation instruments

Scale	Dimension	SPAIK^a^	YSR-K^b^	DIKJ^b^
TALK	Anxiety	0.902**	0.335**	0.426**
REJECT	Anxiety	0.795**	0.380**	0.483**
INTERAC	Deficit	0.760**	0.369**	0.462**
INFORMAT	Deficit	0.676**	0.356**	0.470**
LONELY	Additional scale	0.544**	0.296**	0.384**
TOTAL	Anxiety/deficit	0.885**	0.405**	0.511**

Results are based on the mixed clinical sample (*N* = 85)

*TALK* scale “fear of talking and fear of being in the focus of attention”, *REJECT* scale “fear of rejection”, *INTERAC* scale “interaction deficits”, *INFORMAT* scale “information-processing deficits”, *LONELY* scale “loneliness”, *TOTAL* total scale, *SPAIK* Social Anxiety Disorder and Anxiety Inventory for Children, *YSR-K* Youth Self Report-Short form, *DIKJ* Depression Inventory for Children and Adolescents

** p < 0.01

^a^Correlation by Spearman-Rho (5 % level, two-sided)

^b^Correlation by Kendall (5 % level, two-sided)

When assessing divergent validity, the total scale and all subscales of the SASKO-J were significantly associated with the divergent measures (all *p* ≤ 0.01, see Table 2). However, both anxiety scales showed coefficients below the recommended critical value of *r* < 0.40 for divergent correlations [66] with the YSR-K (*r* = 0.34, *r* = 0.38). This was also true for the subscale INFORMAT of the deficit measures (*r* = 0.36) and the additional scale, LONELY (*r* = 0.29). However, the INTERAC scale of the deficit dimension was barely above the critical threshold (INTERAC *r* = 0.40) as was the total scale (*r* = 0.41). With regard to the DIKJ, all scales of the SASKO-J except LONELY (*r* = 0.38) showed correlations slightly above the recommended cutoff (0.43 ≤ *r* ≤ 0.48).

### Differences between the social anxiety disorder sample and the student sample

The patients with the diagnosis of social anxiety disorder showed significantly higher values in the SASKO-J total scale (*M* = 60.75, *SD* = 22.54, range 8–94) than the students (*M* = 29.85, *SD* = 14.69, range 2–83; *t*(35.77) = −7.52, *p* < 0.001, *r* = 0.64). We also found significant differences with regard to all subscales of the SASKO-J with effect sizes ranging from moderate to high (TALK: *t*(40) = −7.28, *p* < 0.001, *r* = 0.57; REJECT: *t*(40) = −5.78, *p* < 0.001, *r* = 0.48; INFORMAT: *t*(37.66) = −4.67, *p* < 0.001, *r* = 0.43; INTERAC: *U* = 382, *z* = −6.73, *p* < 0.001, *r* = −0.55; LONELY: *U* = 807, *z* = −4.82, *p* < 0.001, *r* = −0.39). The social anxiety disorder patients also showed significantly higher values in the SPAIK (*M* = 31.41, *SD* = 7.32, range 12–45) than the students (*M* = 11.49, *SD* = 7.49, range 0–35; *t*(133) = −12.04, *p* < 0.001, *r* = 0.72).

### Sensitivity and specificity

**Fig. 1:**
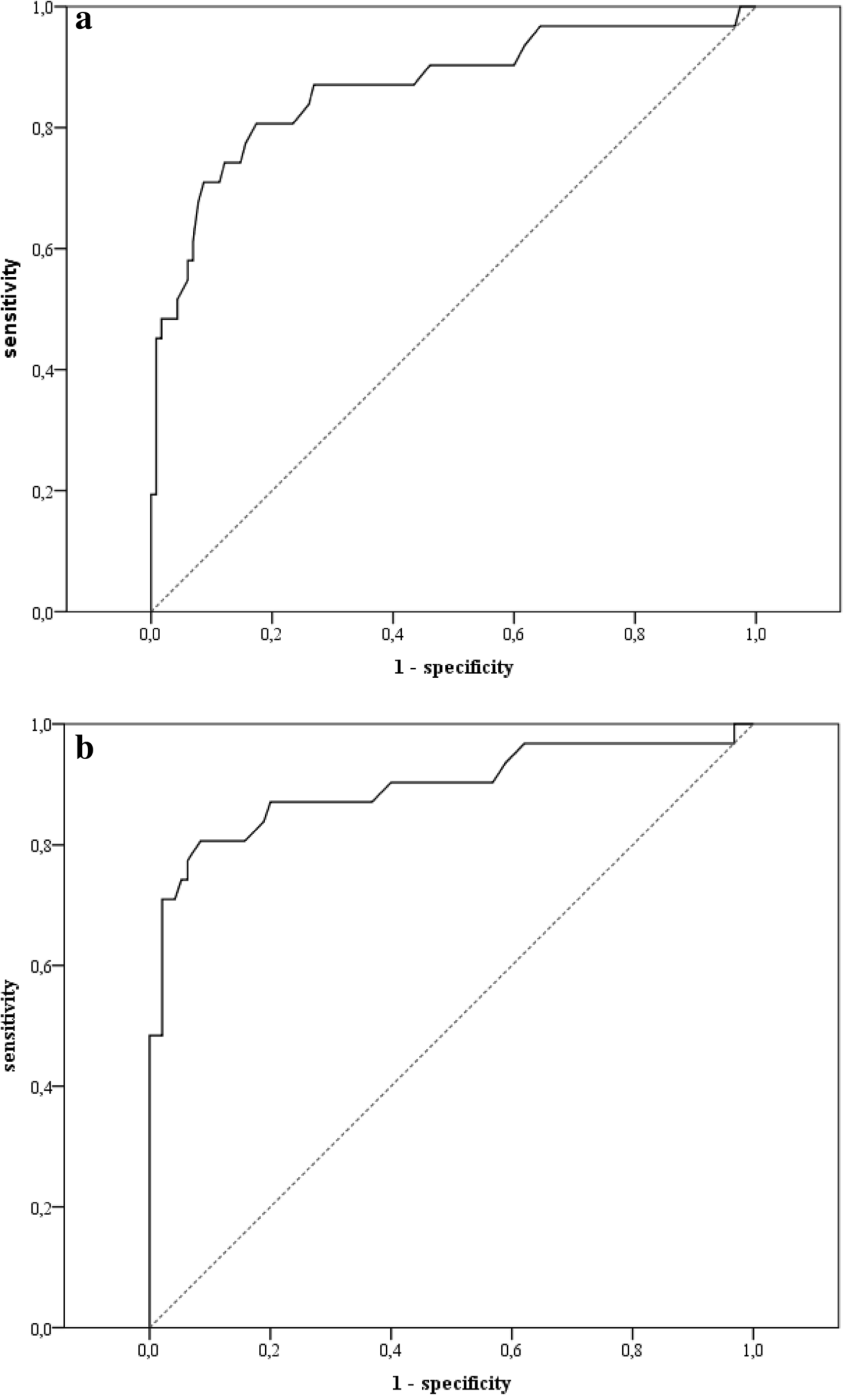
**a** ROC-curve of the SASKO-J total scale with the complete student sample; **b** ROC-curve of the SASKO-J total scale after SPAIK selection of the student sample

Sensitivity and specificity were computed via receiver operating characteristic (ROC) analysis [67]. The main objective was to find a balanced cutoff value that would identify as many adolescents with social anxiety disorder as possible as true positives, and at the same time, indicate youths without social anxiety disorder as true negatives (the students were all considered as having no social anxiety disorder). A high area under the curve (AUC) indicates a high differentiating power [68]. To allow for best comparability with the adult version of the questionnaire, the results for the total scale and for all subscales of the SASKO-J are presented. Figure 1a shows the ROC-curve for the SASKO-J total scale with a good AUC [AUC = 0.866, 95 % CI (0.783, 0.950), *p* < 0.001]. The results for the subscales of the SASKO-J were similar (TALK: AUC = 0.856, *p* < 0.001; REJECT: AUC = 0.804, *p* < 0.001; INFORMAT: AUC = 0.782, *p* < 0.001; INTERAC: AUC = 0.893, *p* < 0.001; LONELY: AUC = 0.774, *p* < 0.001).

On the basis of the coordinates of the ROC-curve and the consideration of the Youden-index [69], a cutoff value of 41.5 was suggested for the total scale (see Table 3). The sensitivity (0.806) and the specificity (0.826) were above the recommended value of 0.70 [46]. The absolute values of true and false decisions for the cutoff at 41.5 are presented in Table 4. With regard to the subscales, the sensitivities were between 0.613 (LONELY) and 0.839 (INFORMAT). The values of specificity ranged between 0.617 (INFORMAT) and 0.870 (TALK; LONELY). The cutoff values were the following: 16.5 (TALK), 11.5 (REJECT), 6.5 (INFORMAT), 8.5 (INTERAC), and 2.5 (LONELY).

**Table 3:** Coordinates of the ROC-Curves of the SASKO-J

	SASKO-J without SPAIK selection^a^			SASKO-J with SPAIK selection^b^		
Value	Sensitivity	1-Specificity	Youden index	Sensitivity	1-specificity	Youden index
39.5	0.806	0.271	0.535	0.806	0.137	0.669
40.5	0.806	0.191	0.615	0.806	0.105	0.701
*41.5*	*0.806*	*0.174*	*0.632*	*0.806*	*0.084*	*0.722*
42.5	0.774	0.157	0.617	0.774	0.063	0.681
43.5	0.742	0.148	0.594	0.742	0.063	0.679

The total scale of the SASKO-J was used for this computation

*SPAIK* Social Anxiety Disorder and Anxiety Inventory for Children

^a^Original sample of students*: N* = 115 students, *N* = 31 patients with social anxiety disorder

^b^Subsample of students who did not reach the cut-off in the SPAIK: *N* = 95 students, *N* = 31 patients with social anxiety disorder diagnosis

**Table 4:** Sensitivity and specificity: Hit rate of the SASKO-J

	Patients (*N* = 31)	Students^a^ (*N* = 115)	Students^b^ (*N* = 95)
Cutoff 41.5	6 false positive (19 %)	95 true negative (83 %)	87 true negative (92 %)
	25 true positive (81 %)	20 false negative (17 %)	8 false negative (8 %)

The total scale of the SASKO-J was used for this computation. All patients had a diagnosis of social anxiety disorder

^a^Original sample of students*: N* = 115 students

^b^Subsample of students who did not reach the cut-off in the SPAIK: *N* = 95 students

The underlying assumption of the above analysis, indicating that none of the students in the sample had a social anxiety disorder diagnosis, is questionable. Thus, we reanalyzed the sample by including only those students who scored under the critical value of 20 in the SPAIK (*n* = 95) [59]. Using this procedure, the AUC was somewhat higher than that in the first analysis [AUC = 0.896, 95 % CI (0.815, 0.975), *p* < 0.001]. Similar improvements were apparent with regard to the subscales (TALK: AUC = 0.888, *p* < 0.001; REJECT: AUC = 0.844, *p* < 0.001; INFORMAT: AUC = 0.805, *p* < 0.001; INTERAC: AUC = 0.913, *p* < 0.001; LONELY: AUC = 0.781, *p* < 0.001). The optimal cutoff-value was again located at 41.5 with equal sensitivity (0.806) but higher specificity (0.916; see Tables 3, 4). The corresponding values of accuracy for the subscales also increased with regard to specificity (0.789–0.926); the cutoff only changed for INFORMAT (from 6.5 to 8.5).

When applying the cutoff of 41.5 to the three samples of our study, the following percentages were found. In the subsample of patients with a social anxiety disorder diagnosis, 81 % were above the cutoff, whereas in the mixed clinical sample, there were only 53 % and in the student sample, only 17 % showed scores above the critical value.

## Discussion

The aim of the present pilot study was (a) to assess the reliability and validity of the SASKO-J in a clinical sample, and (b) to examine its sensitivity and specificity as a diagnostic instrument. As expected, the internal consistencies of the total scale and subscales of the SASKO-J were all very good and provide initial evidence that the questionnaire is a reliable measuring instrument. Although the subscale INFORMAT showed an unsatisfying internal consistency in a sample of students [58], it seems to adequately measure deficits in information processing in our mixed clinical sample of adolescents. The total scale demonstrated a very good internal consistency (α = 0.96). Compared to the established measures we used for validation (SPAIK: α = 0.96; YSR-K and DIKJ: α = 0.88), the SASKO-J has an equal or even a slightly better internal consistency.

The scale inter-correlations are predominantly moderate and comparable to those of the study of Kolbeck [46] with adults. To ensure the intention of the SASKO-J (measuring different aspects of social anxiety disorder), we would have preferred lower inter-correlations between the subscales. However, since the different scales are thought to present different aspects of one underlying disorder, and since anxiety and deficits interact, this result is not surprising [36]. The interaction between those symptoms might play a significant role, particularly within a clinical sample, which could explain why Fernandez Castelao [58] found lower correlations in a student sample.

Regarding the total scale and subscales of the SASKO-J, the convergent validity was supported by the positive association with an established psychometric instrument for the assessment of social anxiety in youths, the SPAIK. When directly comparing the correlations of the anxiety and the deficit scales of the SASKO-J with the SPAIK, in three of the four cases, the correlations with the deficit scales were significantly lower than with the anxiety scales. Kolbeck [46] found similar results in her clinical sample of adults. However, it should be emphasized that there are no adequate instruments available to evaluate the convergent validity of the deficit scales.

In sum, the results regarding the convergent validity suggest that the conceptualization of social anxiety and social deficits as different dimensions of social anxiety disorder—which represents the conceptual basis of the SASKO [36]—is also true for adolescents. However, our findings should only be interpreted as an initial evidence of the validity of the described conceptualization since this study is a pilot. The reasons for the co-occurrence of social competence deficits and symptoms of social anxiety cannot clearly be specified yet. Other explanations for this co-occurrence are also possible, for example, the comorbidity with another disorder or environmental factors (e.g., the lack of experiences). Nonetheless, since anxiety as well as deficits can play an important role in social anxiety disorder, both aspects should be considered to guide intervention strategy decisions.

To evaluate the divergent validity, we computed correlations of the SASKO-J with two different established instruments. In accordance with the hypothesis, the correlation with the YSR-K was low enough to document an adequate divergent validity of the SASKO-J [66]. This finding is especially relevant as the YSR-K, in part, also measures anxiety and social aspects, for example, social insecurity and withdrawal, which overlap with some items of the SASKO-J. Regarding the DIKJ, a somewhat higher correlation was found. This result is not surprising when considering social anxiety and depressive symptoms are not independent constructs. Social anxiety disorder and depressive disorders show high comorbidity [19, 26, 70, 71]. In consideration of these circumstances, the documented associations reflect a sufficiently divergent validity of the SASKO-J.

In sum, the findings provide initial support for very good to good psychometric properties of the SASKO-J when applied in a mixed clinical sample. On this basis, we examined the diagnostic accuracy of the questionnaire in the next step.

As expected, the adolescents with diagnosis of social anxiety disorder showed significantly higher scores in the SASKO-J than the students. The ROC-curve analysis also demonstrated good classification accuracy. It yielded a cutoff of 41.5 resulting in high sensitivity (80 %) and high specificity (82 %), implying high rates of correctly identifying adolescents with social anxiety disorder and of correctly rejecting youths without this disorder. Thus, this pilot study suggests that the SASKO-J can support the clinical diagnostics of social anxiety disorder in the daily routine of therapists as an alternative or additional instrument. In contrast to existing measurements, it also focuses directly on social competence deficits. Moreover, in consideration of the values of sensitivity and specificity of other instruments [57, 59] the discriminant ability of the SASKO-J seems to be promising. After excluding those students from the sample, who according to their scores showed symptoms of social anxiety, the specificity of the SASKO-J increased even more to 91 %, indicating a further reduction of the rate of false positive decisions. However, it should be stressed that this exclusion may have resulted in an overestimation of sensitivity and specificity due to an artificially low ceiling of values in the sample of students. In sum, the findings of Kolbeck and Maß [36] with regard to the diagnostic accuracy are similar to that of the SASKO-J.

Since the total scale does not allow a statement about the individual constellation of anxiety and deficits, the cutoff values of the subscales should also be considered by clinicians for their therapy decisions [36]. This possibility of differentiated information is the main characteristic of the SASKO-J. The adequate assessment of different symptoms and deficits is especially important in adolescence since they can hinder important social interactions in this developmental stage [7, 9]. In addition, social anxiety disorder and accompanying problems in adolescence can lead to problems with peers and can finally result in loneliness. Loneliness in turn might play an especially significant role in the maintenance and chronicity of social anxiety disorder [9] and thus, should also be considered with regard to therapeutic decisions.

## Strengths and limitations

One of the biggest strengths of this pilot study is that it provides first information about the psychometric qualities of a new diagnostic instrument. This instrument sets itself apart from existing social anxiety disorder measures by also focusing on social deficits. A further strength can be seen in the characteristics of the clinical samples. Since adolescents were recruited from different types of institutions in various cities, and differed in their comorbidities, the diversity of patients is well represented. Thus, the generalization of results to German adolescents (aged 12–19 years) with psychiatric diagnoses is possible.

The study also has limitations. The majority of youths from the mixed clinical sample did not have a diagnosis of social anxiety disorder but had other psychiatric disorders. Since the social anxiety disorder sample was too small, an adequate evaluation of psychometric properties in a “pure” social anxiety disorder sample was not possible. Thus, the generalization to individuals with social anxiety disorder is limited. Moreover, the study should be considered as a pilot study that provides only initial support for reliability and validity. Also, the quality of given diagnoses and with this, the results of the ROC curve and group differences, must be interpreted with caution since only half of the therapists based them on a clinical interview. Due to temporal and organizational reasons, it was not possible for the researchers to perform diagnostic interviews with the student sample. However, we reduced the possibility that many students were actually “hidden” social anxiety disorder patients by reanalyzing the ROC-curve after eliminating those showing high scores in the SPAIK. In addition, the duration of therapy and the fact that some patients were taking drugs could have influenced the level of symptoms and thus, the results. The results regarding convergent validity of the deficit scales must be interpreted with caution as no measures were used that examined social competence deficits.

## Clinical implications and future research

The SASKO-J was developed to support the clinical diagnostics and the planning of therapy. Since different constellations of social anxiety and social deficits may implicate a different therapeutic approach, the explicit clarification of symptoms at an early point in treatment is desirable. It can offer recommendations for the focus of therapy, e.g., regarding the usefulness of specific social competence training, especially when considering that deficits in social skills and social cognition are related to a higher level of symptoms and adverse development of the disorder over time [72].

On the basis of the present results, future studies should evaluate the psychometric properties of the SASKO-J in a social anxiety disorder sample. In this context, the diagnostic accuracy should also be analyzed using a clinical sample in addition to that of a student sample and it should be assessed whether social anxiety disorder can be distinguished from other anxiety disorders. Moreover, the retest-reliability and the sensitivity for changes in symptomatology should be examined to document the questionnaire’s utility for the therapeutic process.

## Conclusion

Past research has only marginally focused on social deficits within the context of social anxiety disorder; however, clear suggestions to clarify their existence can be found [46–48]. Nevertheless, appropriate diagnostic instruments have been missing [73]. Different diagnostic means, such as the observation of behavior in role play and behavioral experiments, provide information about the visual social performance but they do not offer insight into conscious and unconscious processes of social perception and cognition or information processing. Since these latter aspects are also important aspects of social competence deficits [46], they should not be neglected during the diagnostic process. These important aspects are part of the SASKO-J. Thus, the SASKO-J can be seen as a first step to close the gap between the theoretical consideration of two separate dimensions of social anxiety disorder and its practical implementation in the diagnostic and therapeutic process. The results of the present pilot study and the previous study [58] are promising but they offer only initial evidence to designate this questionnaire as a reliable, valid, and highly differentiating instrument. To draw a final conclusion, more comprehensive evidence is needed.

## Footnotes

1 Since it was very difficult to recruit adolescents with social anxiety disorder, the corresponding sample was rather small. To obtain meaningful results, a mixed clinical sample was therefore used for analyses of reliability and validity.

2 In Germany, students attend grammar school from fifth to twelfth grade and finish with qualifications for university admission or matriculation. Students who attend a secondary general school do so from fifth to tenth grade and finish with the General Certificate of Secondary Education, which qualifies them for further education or job training. Graduation from a secondary modern school (grade 5 to grade 9) results in the Certificate of Secondary Education that allows for job training. A comprehensive school combines these types of schooling.

3 The results of reliability and validity refer to the mixed clinical sample (*N* = 85). For the computation of mean differences and the examination of sensitivity and specificity, the social anxiety disorder sample (*N* = 31) and the student sample (*N* = 115) were used.

4 Since the focus of the present paper was on the reliability and validity of the SASKO-J in a clinical sample, the results concerning the factor structure and the item characteristics are not presented here but can be requested from the corresponding author.
